# Complete plastid genome sequences of *Drimys, Liriodendron*, and *Piper*: implications for the phylogenetic relationships of magnoliids

**DOI:** 10.1186/1471-2148-6-77

**Published:** 2006-10-04

**Authors:** Zhengqiu Cai, Cynthia Penaflor, Jennifer V Kuehl, James Leebens-Mack, John E Carlson, Claude W dePamphilis, Jeffrey L Boore, Robert K Jansen

**Affiliations:** 1 Section of Integrative Biology and Institute of Cellular and Molecular Biology, Patterson Laboratories 141, University of Texas, Austin, TX 78712, USA; 2 Biology Department, 373 WIDB, Brigham Young University, Provo, UT 84602, USA; 3 DOE Joint Genome Institute and Lawrence Berkeley National Laboratory, Walnut Creek, CA 94598, USA; 4 Department of Plant Biology, University of Georgia, Athens GA, USA; 5 School of Forest Resources and Huck Institutes of the Life Sciences, The Pennsylvania State University, University Park, PA 16802, USA; 6 Department of Biology, Huck Institutes of the Life Sciences, The Pennsylvania State University, University Park, PA 16802, USA

## Abstract

**Background:**

The magnoliids with four orders, 19 families, and 8,500 species represent one of the largest clades of early diverging angiosperms. Although several recent angiosperm phylogenetic analyses supported the monophyly of magnoliids and suggested relationships among the orders, the limited number of genes examined resulted in only weak support, and these issues remain controversial. Furthermore, considerable incongruence resulted in phylogenetic reconstructions supporting three different sets of relationships among magnoliids and the two large angiosperm clades, monocots and eudicots. We sequenced the plastid genomes of three magnoliids, *Drimys *(Canellales), *Liriodendron *(Magnoliales), and *Piper *(Piperales), and used these data in combination with 32 other angiosperm plastid genomes to assess phylogenetic relationships among magnoliids and to examine patterns of variation of GC content.

**Results:**

The *Drimys*, *Liriodendron*, and *Piper *plastid genomes are very similar in size at 160,604, 159,886 bp, and 160,624 bp, respectively. Gene content and order are nearly identical to many other unrearranged angiosperm plastid genomes, including *Calycanthus*, the other published magnoliid genome. Overall GC content ranges from 34–39%, and coding regions have a substantially higher GC content than non-coding regions. Among protein-coding genes, GC content varies by codon position with 1st codon > 2nd codon > 3rd codon, and it varies by functional group with photosynthetic genes having the highest percentage and NADH genes the lowest. Phylogenetic analyses using parsimony and likelihood methods and sequences of 61 protein-coding genes provided strong support for the monophyly of magnoliids and two strongly supported groups were identified, the Canellales/Piperales and the Laurales/Magnoliales. Strong support is reported for monocots and eudicots as sister clades with magnoliids diverging before the monocot-eudicot split. The trees also provided moderate or strong support for the position of *Amborella *as sister to a clade including all other angiosperms.

**Conclusion:**

Evolutionary comparisons of three new magnoliid plastid genome sequences, combined with other published angiosperm genomes, confirm that GC content is unevenly distributed across the genome by location, codon position, and functional group. Furthermore, phylogenetic analyses provide the strongest support so far for the hypothesis that the magnoliids are sister to a large clade that includes both monocots and eudicots.

## Background

Phylogenetic relationships among basal angiosperms have been debated for over 100 years since Darwin [1] identified the origin and rapid diversification of flowering plants as an "abominable mystery". The difficulty of resolving these relationships is likely due to the rapid radiation of angiosperms. During the past decade there has been considerable interest in estimating phylogenetic relationships based on single or multiple genes to resolve the basal radiation of flowering plants [2-19]. Recently, completely sequenced plastid genomes have been used to estimate relationships among basal angiosperms [9-11,18,19] and comprehensive analyses of these data concur with earlier studies resolving either *Amborella *or *Amborella *plus the Nymphaelales as the basal-most clade, sister to all other angiosperms [2-8], [12-20]. Although the whole plastid genome approach has enhanced our understanding of basal angiosperm relationships, issues of taxon sampling and methods of phylogenetic analysis have generated considerable controversy regarding the efficacy of this approach [13,18-23]. One of the major limitations of this approach is the paucity of plastid genome sequences from early diverging lineages, especially the magnoliids, which are currently represented only by *Calycanthus *[24].

With four orders, 19 families, and approximately 8,500 species, the magnoliids comprise the largest clade of early diverging angiosperms [25]. Recent single and multigene trees have provided only weak to moderate support for the monophyly of this clade [7,14,15,26-28]. One notable exception is the phylogenetic estimate of Qiu et al. [17] based on eight plastid, mitochondrial, and nuclear genes and 162 taxa representing all of the major lineages of gymnosperms and angiosperms. This eight-gene tree provides the first strong support for the monophyly of the four orders of magnoliids, and for sister group relationships between the Canellales/Piperales and Laurales/Magnoliales.

One of the most important remaining phylogenetic issues regarding angiosperms is the relationship of magnoliids to the other major clades, including the monocots and eudicots. All three possible relationships among these major angiosperm clades have been generated based on single and multiple gene trees, but none receives strong support in any of these studies [reviewed in [25]]. The three-gene phylogenetic tree of Soltis *et al*. [27] placed the magnoliids and monocots in a clade that was sister to the eudicots. Support for the monophyly of this group, which was referred to as eumagnoliids, was weak with a jackknife value of only 56%. This same topology was generated in Bayesian analyses using the plastid gene *matK*, with a parsimony bootstrap value of 78% and a Bayesian posterior probability of 0.73 [29]. Two multi-gene molecular phylogenetic studies identified the magnoliids sister to the eudicots. The 11-gene MP trees in Zanis *et al*. [7] provided only weak support (56% bootstrap value) for the sister group relationship between magnoliids and eudicots, and in the 9-gene analyses of Qiu *et al. *[16] support for this same relationship increased to 78% in ML trees. Finally, a third possible resolution of relationships among magnoliids, monocots, and eudicots was recovered in two other studies based on phytochrome genes [3] and 17 plastid genes [6]. These studies suggested that magnoliids were sister to a clade that included monocots and eudicots, however, support for this relationship had only weak or moderate bootstrap support (< 50% in Mathews and Donoghue [3] and 76 or 83% in Graham and Olmstead [6]). Thus, despite intensive efforts during the past 10 years relationships among magnoliids, monocots, and eudicots remain problematic.

In this paper, we report on the complete sequences of three magnoliid plastid genomes (*Drimys, Liriodendron*, and *Piper*). We characterize the organization of two of these genomes, including the most comprehensive comparisons of GC content among completely sequenced plastid genomes. Furthermore, the results of phylogenetic analyses of DNA sequences of 61 genes for 35 taxa, including 33 angiosperms and two gymnosperm outgroups provide new evidence for resolving relationships among angiosperms with an emphasis on relative positions of magnoliids, monocots and eudicots. These results have implications for our understanding of floral evolution in the angiosperms [e.g., [30]].

## Results

### Size, gene content, order and organization of the *Drimys *and *Piper *plastid genomes

We have sequenced the plastid genomes of three genera of magnoliids, *Drimys, Liriodendron*, and *Piper*. In this paper, we only characterize the genome of two of these, *Drimys *and *Piper*, and we use 61 protein-coding genes from all three for the phylogenetic analyses. The genome sequence for *Liriodendron *will be described in a future paper on the utility of 454 sequencing [31] for plastid genomes (J. E. Carlson et al. in progress).

**Figure 1:**
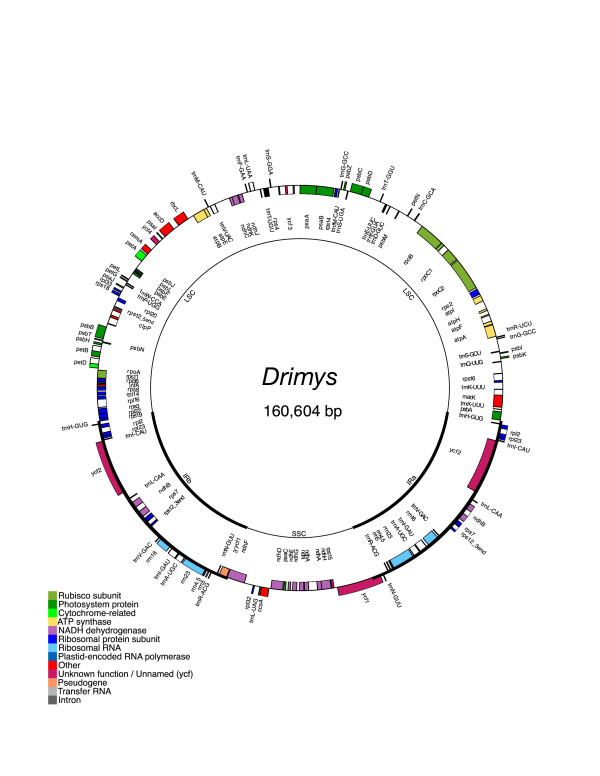
Gene map of the *Drimys granatensis *plastid genome. The thick lines indicate the extent of the inverted repeats (IRa and IRb), which separate the genome into small (SSC) and large (LSC) single copy regions. Genes on the outside of the map are transcribed in the clockwise direction and genes on the inside of the map are transcribed in the counterclockwise direction.

**Figure 2:**
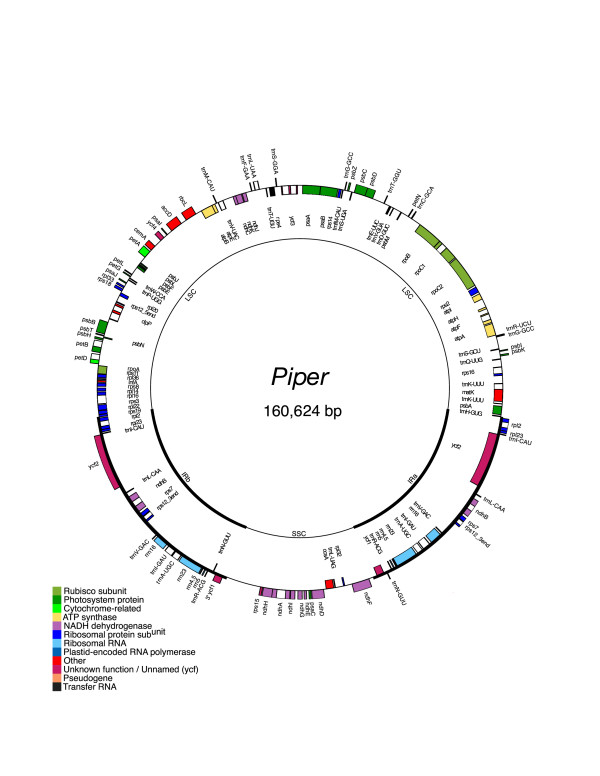
Gene map of the *Piper coenoclatum *plastid genome. The thick lines indicate the extent of the inverted repeats (IRa and IRb), which separate the genome into small (SSC) and large (LSC) single copy regions. Genes on the outside of the map are transcribed in the clockwise direction and genes on the inside of the map are transcribed in the counterclockwise direction.

The sizes of the *Drimys *and *Piper *plastid genomes are nearly identical at 160,604 and 160,624 bp, respectively (Figs. 1, 2), and within a kb of the *Liriodendron *plastid genome size (159,886 bp; J. E. Carlson et al. in progress). The genomes include a pair of inverted repeats of 26,649 bp (*Drimys*) and 27,039 bp (*Piper*), separated by a small single copy region of 18,621 bp (*Drimys*) and 18,878 bp (*Piper*) and a large single copy region 88,685 bp (*Drimys*) and 87,668 bp (*Piper*). The *Drimys *IR has expanded on the IRa side to duplicate *trnH*-*gug*. This expansion has not increased the overall size of the IR in *Drimys *because two of the genes in the IR of *Drimys *are shorter than they are in *Piper *(*ycf2 *is 6909 and 6945 and *ndhB *is 1533 and 1686 in *Drimys *and *Piper*, respectively).

The *Drimys *and *Piper *plastid genomes contain 113 different genes, and 18 (*Drimys*) and 17 (*Piper*) of these are duplicated in the IR, giving a total of 130–131 genes (Figs. 1, 2, Table 1). There are only two differences in gene content between these two magnoliid genomes; one is due to the duplication of *trnH-gug *in *Drimys *and the second is that *ycf1 *appears to be a pseudogene in *Piper*, since it has an internal stop codon that results in a truncated gene that is only 927 bp long (versus 5,574 bp in *Drimys*). Eighteen genes contain introns, 15 of which contain one intron and three (*clpP, rps12*, and *ycf3*) with two introns (Table 1). There are 30 distinct tRNAs, and 8 and 7 of these are duplicated in the IR of *Drimys *and *Piper*, respectively. The genomes consist of 50.12% (*Drimys*) and 48.36% (*Piper*) protein-coding genes, 7.38% (*Drimys*) and 7.34% (*Piper*) RNA genes, and 42.5% (*Drimys*) and 44.3% (*Piper*) non-coding regions (intergenic spacers and introns). Gene order is identical in the magnoliid plastid genomes sequenced from *Drimys, Piper, Liriodendron *(J. E. Carlson et al. in progress), and *Calycanthus *[24], and those of most angiosperms, including tobacco.

**Table 1:** Comparison of major features of magnoliid plastid genomes

	*Calycanthus*	*Drimys*	*Piper*
Size (bp)	153,337	160,604	160,624
LSC length (bp)	86,948	88,685	87,668
SSC length (bp)	19,799	18,621	18,878
IR length (bp)	23,295	26,649	27,039
Number of genes	133 (115)	131 (113)	130 (113)
Number of gene duplicated in IR	18	18	17
Number of genes with introns (with 2 introns)	18 (3)	18 (3)	18 (3)

### GC content

**Figure 3:**
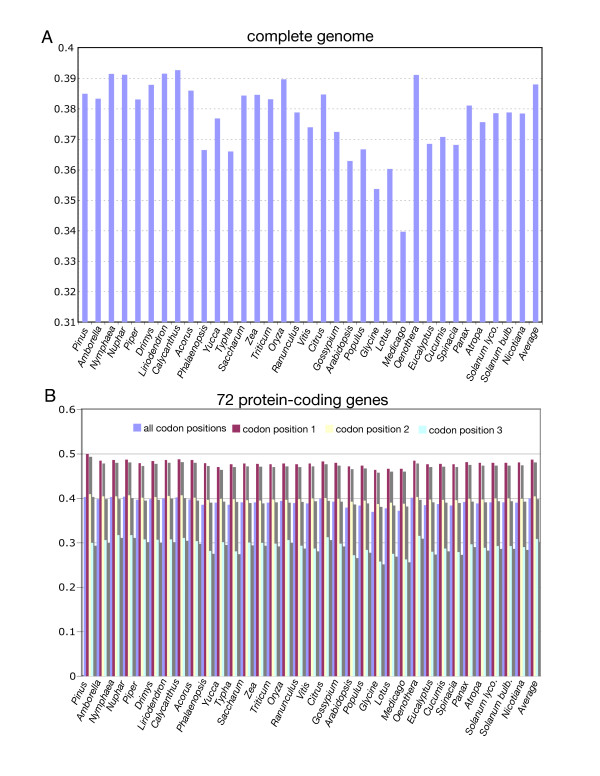
Histogram of GC content for 34 seed plant plastid genomes, including the gymnosperm *Pinus *and 33 angiosperms (see Table 2 for list of genomes). Taxa are arranged phylogenetically following tree in Fig. 8. **A. **Overall GC content of complete genomes. **B. **GC content for 66 protein-coding genes, including average value for all codon positions, followed by values for the 1st, 2nd, and 3rd codon positions, respectively.

**Figure 4:**
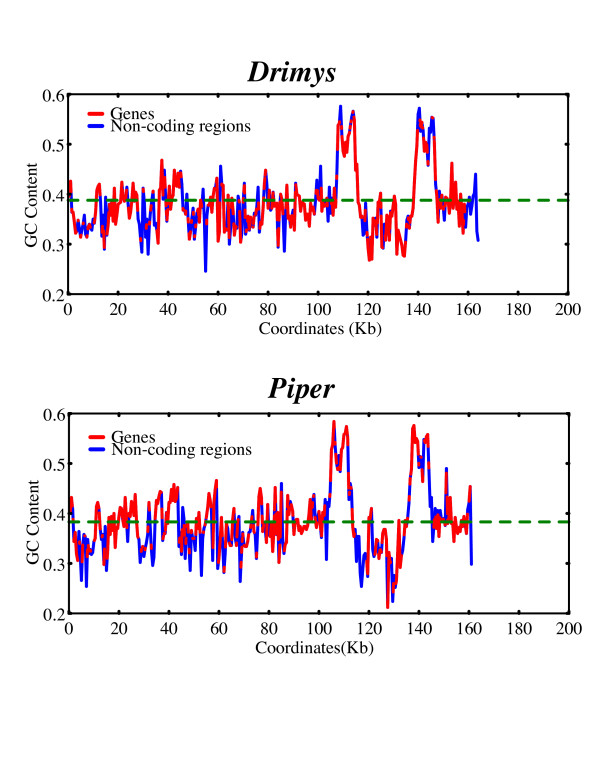
Graphs of GC content plotted over the entire plastid genomes of *Drimys *and *Piper*. X axis represents the proportion of GC content between 0 and 1 and the Y axis gives the coordinates in kb for the genomes. Coding and non-coding regions are indicated in blue and red, respectively. The green dashed line indicates that average GC content for the entire genome.

**Figure 5:**
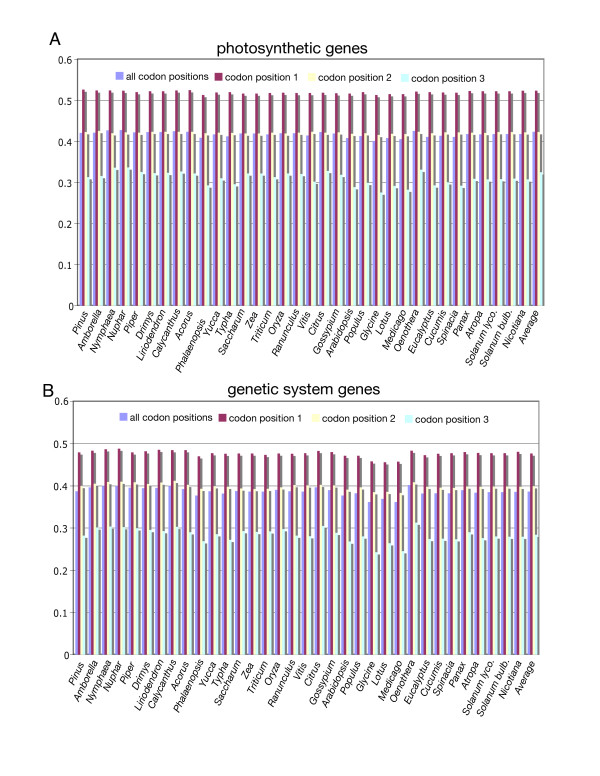
Histogram of GC content for photosynthetic and genetic system genes for 34 seed plant plastid genomes (see Table 2 for list of genomes). Taxa are arranged phylogenetically following tree in Fig. 8. GC content includes average value for all codon positions, followed by values for the 1st, 2nd, and 3rd codon positions, respectively. **A. **GC content for 33 photosynthetic genes. **B. **GC content for 22 genetic system genes.

**Figure 6:**
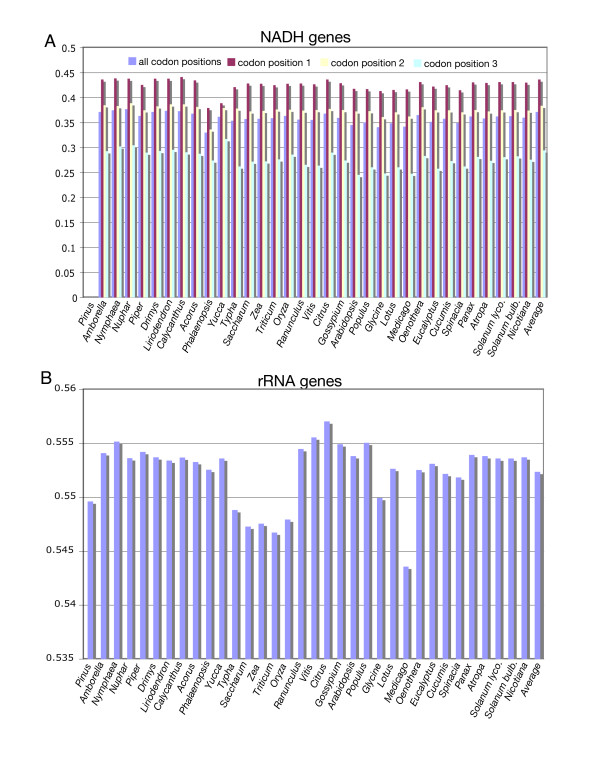
Histogram of GC content for NADH and rRNA genes for 34 seed plant plastid genomes (see Table 2 for list of genomes). Taxa are arranged phylogenetically following tree in Fig. 8. **A. **GC content for 11 NADH genes, which includes average value for all codon positions, followed by values for the 1st, 2nd, and 3rd codon positions, respectively. **B. **GC content for four rRNA genes.

**Figure 7:**
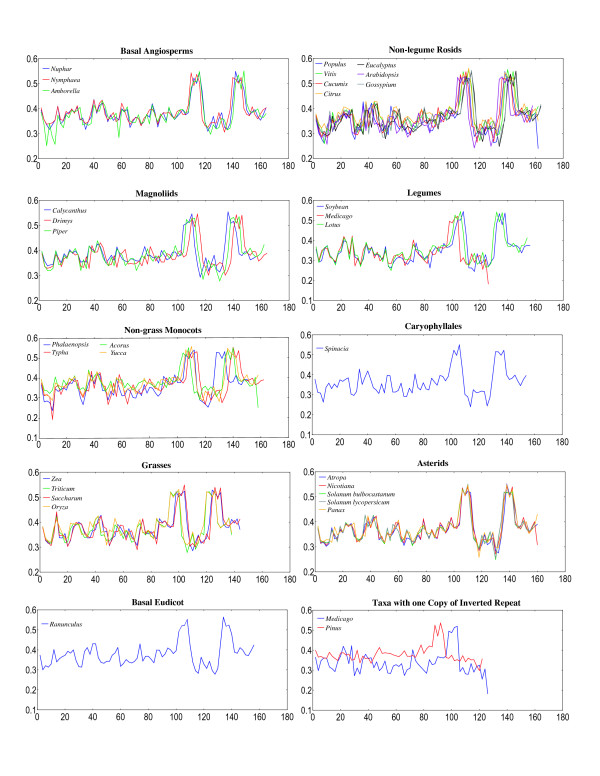
Graphs of GC content plotted over the entire plastid genomes of 34 seed plants. The graphs are organized by genomes with the same gene order and by clade in the phylogenetic trees in Figures 8 and 9. X axis represents the proportion of GC content between 0 and 1 and the Y axis gives the coordinates in kb for the genomes.

The overall GC content of the *Piper *and *Drimys *plastid genomes is similar, 38.31% and 38.79% respectively. These values are within the range of 34–39% GC content but slightly higher than that of the average for 35 plastid genomes representing all currently available angiosperms and one gymnosperm *Pinus *(Fig. 3A). In some cases, overall GC content correlates with phylogenetic position: early diverging lineages (i.e., *Amborella*, Nymphaeales, magnoliids) tend to have a higher GC content and legumes and non-grass monocots have lower values (Fig. 3A). GC content is not uniformly distributed across the plastid genome (Figs. 3, 4, 5, 6, 7). In general, GC content is higher in coding regions than in non-coding regions (i.e., intergenic spacers and introns) (Fig. 4). This pattern is also supported by the observation that GC content of protein-coding genes is higher than the overall GC content for the complete genomes (compare Figs 3A,B). A t test indicated that this pattern is statistically significant at p < 0.01. GC content also varies by codon position with the 1st codon > 2nd codon > 3rd codon (Figs. 5A,B, 6A). GC content was also compared by partitioning protein-coding genes into three functional groups (Figs 3A, 5A,B, 6A). This comparison demonstrates that the percent of GC for all three codon positions is highest in photosynthesis genes, followed by genetic system genes, and lowest in NADH genes. Statistical tests using ANOVA demonstrated that differences in GC content by codon positions and functional groups are significant at p < 0.01. GC content is similar among all genomes (Fig. 7) even in taxa that have different gene orders (*i.e*., grasses and legumes). The IR regions have higher GC content and the SSC has the lowest. The much higher GC content in the IR is due to the presence of rRNA genes (Fig. 6B). The lower GC content in the SSC is due to the presence of eight of the 11 NADH genes, which have a lower GC content than photosynthetic and genetic system genes (Figs. 5A,B, 6A). This genome wide pattern of GC content is maintained even when one copy of the IR is lost (bottom right panel in Fig. 7).

### Phylogenetic analyses

Our phylogenetic data set included 61 protein-coding genes for 35 taxa (Table 1), including 33 angiosperms and two gymnosperm outgroups (*Pinus *and *Ginkgo*). The data set comprised 45,879 nucleotide positions but when the gaps were excluded there were 39,378 characters.

**Figure 8:**
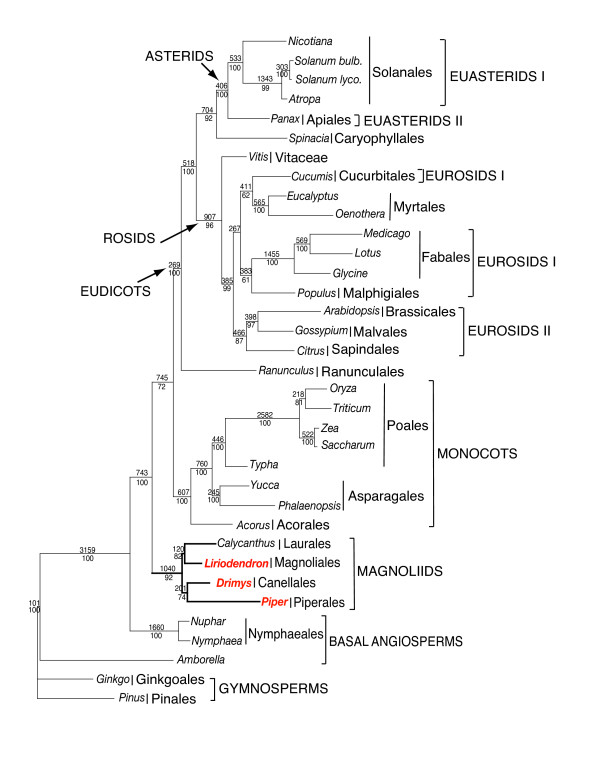
Phylogenetic tree of 35-taxon data set based on 61 plastid protein-coding genes using maximum parsimony. The tree has a length of 61,095, a consistency index of 0.41 (excluding uninformative characters) and a retention index of 0.57. Numbers at each node are bootstrap support values. Numbers above node indicate number of changes along each branch and numbers below nodes are bootstrap support values. Ordinal and higher level group names follow APG II [93]. Taxa in red are the three new genomes reported in this paper.

**Figure 9:**
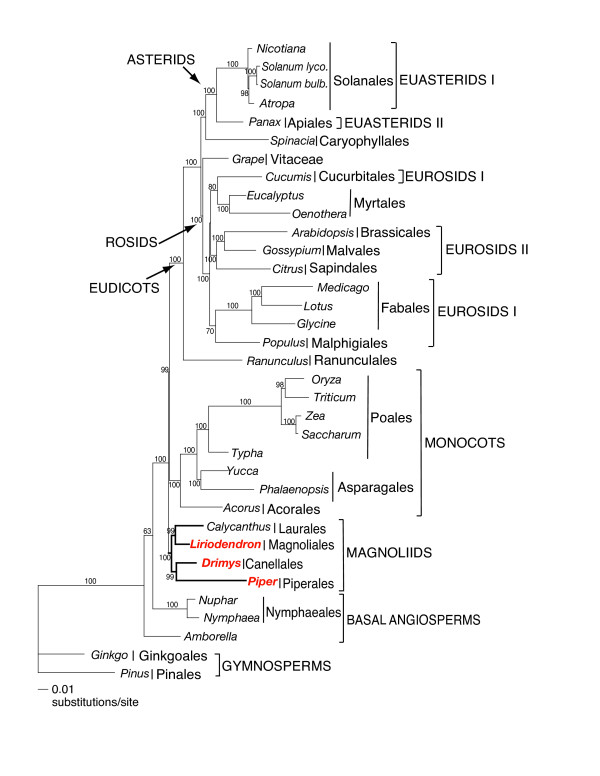
Phylogenetic tree of 35-taxon data set based on 61 plastid protein-coding genes using maximum likelihood. The single ML tree has an ML value of – lnL = 342478.92. Numbers at nodes are bootstrap support values ≥ 50%. Scale at base of tree indicates the number of base substitutions. Ordinal and higher level group names follow APG II [93]. Taxa in red are the three new genomes reported in this paper.

Maximum Parsimony (MP) analyses resulted in a single, fully resolved tree with a length of 61,095, a consistency index of 0.41 (excluding uninformative characters), and a retention index of 0.57 (Fig. 8). Bootstrap analyses indicated that 22 of the 32 nodes were supported by values ≥ 95% and 18 of these had a bootstrap value of 100%. Of the remaining 10 nodes, five had bootstrap values between 80–95%. Maximum likelihood (ML) analysis resulted in a single tree with – lnL = 342478.92 (Fig. 9). ML bootstrap values also were also high, with values of ≥ 95% for 28 of the 32 nodes and 100% for 23 these nodes. The ML and MP trees had similar topologies. Both trees indicate that *Amborella *alone forms the earliest diverging angiosperm, however, support for this placement is much higher in the MP tree (100%) than the ML tree (63%). The next most basal clade includes the Nymphaeales (*Nuphar *and *Nymphaea*) and support for this relationship is 100% in both MP and ML trees. Recent phylogenetic trees based on complete plastid genome sequences [11,18,19] have highlighted the difficulty of resolving the relative position of *Amborella *and the Nymphaeales. Two alternative hypotheses that have received the most support are: *Amborella *as the earliest diverging lineage of angiosperms, or *Amborella *and the Nymphaeales forming a clade sister to all remaining extant angiosperms. In a previous study using 61 plastid genes (see Fig. 4 in [18]) the first hypothesis was strongly supported (100%) in parsimony trees and the second hypothesis received only moderate support (63%) in ML trees. Both MP and ML trees support the basal position of *Amborella *alone in our expanded taxon sampling. We performed a SH test [32] to determine if the *Amborella*/Nymphaeales basal hypothesis is a reasonable alternative to the ML and MP trees that support the *Amborella *basal topology. The ML score for the alternative topology was -ln L = 339758.53918 versus 339753.32204 for the best ML tree. The difference in the -ln L was 5.21714 with a p = 0.303. Thus, the *Amborella*/Nymphaeales basal hypothesis could not be rejected by the SH test, indicating that identification of the most basal angiosperm lineage remains unresolved even with the addition of three magnoliid genomes.

Relationships among most other major angiosperm clades are congruent in the MP and ML trees. Monophyly of the magnoliids is strongly supported with 92 (MP) or 100% (ML) bootstrap values. Within magnoliids there are two well-supported clades, one including the Canellales/Piperales with 74 (MP) or 99 (ML)% bootstrap support, and a second including the Laurales/Magnoliales with 82 (MP) or 99 (ML)% bootstrap support. The magnoliid clade forms a sister group to a large clade that includes the monocots and eudicots. Support for the sister relationship of magnoliids to the remaining angiosperms is moderate (72% in MP tree, Fig. 8) or strong (99% in ML tree, Fig. 9).

Support for the monophyly of monocots and eudicots is strong with 100% bootstrap values, and relationships among the monocots is identical in both analyses. The Ranunculales occupy the earliest diverging lineage among eudicots, and they are sister to two major, strongly supported clades, the rosids and Caryophyllales/asterids. There is strong support for the sister group relationship between the Caryophyllales and asterids (92% in MP and 100% in ML). Both MP and ML trees provide strong support for the placement of *Vitis *as the earliest diverging lineage within the rosids. The only remaining incongruence between MP and ML trees is found within the rosids. In both analyses, eurosids I are not monophyletic, although support for relationships among the five representatives of this clade to the eurosid II and Myrtales clades is not strong.

## Discussion

### Genome organization and evolution of GC content

The organization of the *Drimys *and *Piper *genomes with two copies of an IR separating the SSC and LSC regions is identical to most sequenced angiosperm plastid genomes [reviewed in [33]]. The sizes of the genome at 160,604 and 160,624 bp, respectively are similar to the each other and *Liriodendron*, but larger than the only other sequenced magnoliid genome (*Calycanthus*, 153,337, 23, Table 1). Most of this size difference is due to the larger size of the IR in *Drimys *(26,649 bp) and *Piper *(27,039 bp) relative to *Calycanthus *(23,295 bp), although some is also due to the larger LSC region (Table 1). Expansion and contraction of the IR is a common phenomenon in land plant plastid genomes [34] with the IR ranging in size from 9,589 bp in the moss *Physcomitrella *[35] to 75,741 bp in the highly rearranged angiosperm genome of *Pelargonium *[36,37]. Among angiosperms the IR generally ranges in size between 20–27 kb, and the magnoliid genomes except for *Calycanthus *are at the high end of that range.

Gene order of the magnoliid plastid genomes is identical to tobacco and many other unrearranged angiosperm plastid genomes. There are a few differences in gene content and these can be explained by two phenomena. The first concerns differences in the annotation of two genes in these genomes. Two putative genes (*ACRS *and *ycf15*) in *Calycanthus *were not annotated in *Drimys *and *Piper *because several recent studies indicated that they are not functional plastid genes. The sequence of *ycf15 *has been shown to be highly variable among angiosperms, with conserved motifs at the 5' and 3' ends and an intervening sequence that makes it a pseudogene [38,39]. An examination of *ycf15 *transcripts in spinach indicated that is not a functional protein-coding gene [38]. More recent sequence comparisons of *ycf15 *in other plastid genomes also supported the conclusion that this putative gene is not functional [9,39]. The ACRS gene was identified by Goremykin et al. [24] in *Calycanthus *based on its very high sequence identity with the mitochondrial ACR-toxin sensitivity (*ACRS*) gene of *Citrus jambhiri *[40]. This conserved sequence has been identified (as *ycf68*) in a number of plastid genomes, however, in cases where it has been critically examined the presence of internal stop codons indicates that this is a pseudogene. The second explanation for gene content differences among the three magnoliid genomes is caused by the expansion of the IR in *Piper*, which results in the duplication of *trnH*. Small expansions of the IR boundary are common in plastid genomes [34] resulting in duplications of genes at the IR/SC boundaries. The duplication of *trnH *in *Piper *is shared with *Nuphar*, a member of the Nymphaeales (L. Raubeson et al. unpublished). This expansion of the IR to duplicate *trnH *has clearly happened independently in *Piper *and *Nuphar *since none of the other basal angiosperms or magnoliids have this duplication.

Examination of GC content in 34 seed plant plastid genomes reveals several clear patterns. GC content for the complete genomes ranges between 34–39% (Fig. 3A), confirming previous observations that plastid genomes are in general AT rich [24,39,41-47]. The uneven distribution of GC content over the plastid genome is also very evident, and there are several explanations for this pattern. First, there is a clear bias for the coding regions to have a significantly higher GC content than non-coding regions (Figs. 3, 4), which again confirms previous observations based on comparisons of many fewer genomes [24]. Second, there is an uneven distribution of GC content by regions of the genome with the highest GC content in the IR and the lowest in the SSC (Figs. 4, 7). The higher GC content in the IR can be attributed to the presence of the four rRNA genes in this region, which have the highest GC content of any coding regions (Fig. 6B). This higher GC content in the IR region is maintained even when one copy of the IR is lost as in *Medicago *and *Pinus *(Fig 7, bottom right panel). The lower GC content in the SSC region is due to the presence of 8 of the 11 NADH genes, which have the lowest GC content of any of the classes of genes compared (Figs. 5, 6). Third, GC content varies by functional groups of genes. Among protein genes, GC content is highest for photosynthetic genes, lowest for NADH genes, with genetic system genes having intermediate values. This same pattern was observed by Shimada and Sugiura [41] in comparisons of the first three sequenced land plant plastid genomes.

Differences in GC content were also observed by codon position in protein-coding genes (Figs. 3B, 5A,B, 6A). For each of the three classes of genes (photosynthetic, genetic system, and NADH) the third position in the codon has a significant AT bias. This pattern has been observed previously [41,45,46,48], and it has been attributed to codon bias. Previous studies have demonstrated that there is a strong A+T bias in the third codon position for plastid genes [45,46,48]. This is in contrast to a GC bias in codon usage for nuclear genes in plants [48]. Several studies have examined codon usage of plastid genes to attempt to determine if these biases can be attributed to nucleotide compositional bias, selection for translational efficiency, or a balance among mutational biases, natural selection, and genetic drift [49-53]. All of these studies have been limited to examining a single or few genes, and they have been constrained by the limited sampling of complete genome sequences for taking variation in GC content into account. Our comparisons of GC content variation for a wide diversity of angiosperm lineages provide a rich source of information for future investigations of the relationship between GC content and codon usage bias.

### Phylogenetic implications

The debate concerning the identity of the most basal angiosperm lineage continues even though numerous molecular phylogenetic studies of angiosperms have been conducted over the past 15 years [3,4,6,8-11], [14-20], [26-29], [54-66]. Several issues have confounded the resolution of relationships among basal angiosperms, including long branch attraction associated with sparse taxon density and poor taxon sampling, and conflict among trees obtained using different phylogenetic methodologies [4,6,7,11,13,18-20,67]. Most recent studies agree that *Amborella *and the Nymphaeales represent the earliest diverging angiosperm lineages [3,4,6-8], [14-20], [26-29], [57-66]. The most recent multi-gene phylogenetic reconstructions based on nine gene sequences from the plastid, mitochondrial, and nuclear genomes [17] generate trees supporting each of these two hypotheses depending on the method of phylogenetic analysis and the genes included. Trees generated from plastid genes supported the *Amborella *basal hypothesis, whereas mitochondrial genes supported the *Amborella *+ Nymphaeales hypothesis. Furthermore, MP analyses tended to support the *Amborella *basal hypothesis and ML analyses supported *Amborella *+ Nymphaeales. A similar set of relationships was also observed in recent phylogenetic studies using sequences of 61 genes from completely sequenced plastid genomes [18,19,67]. In these studies, MP trees placed *Amborella *alone as the basal most angiosperm with strong support and ML trees placed *Amborella *+ Nymphaeales at the base with moderate support. These differences were attributed to rapid diversification and the lack of extant lineages that could be used to cut the length of branches leading to *Amborella *and the most recent common ancestor of lineages within the Nymphaeales.

Our phylogenetic analyses include three additional magnoliids from three different orders. Both MP and ML trees (Figs. 8, 9) support *Amborella *alone as the earliest diverging lineage of angiosperms. Support for this relationship is very strong in MP trees (100% bootstrap) and weak (63%) in ML trees. However, a SH test that constrained *Amborella *+ Nymphaeales in a basal position indicated that the two hypotheses of basal angiosperm relationships are not significantly different. Thus, although both MP and ML analyses including the three additional magnoliid taxa support *Amborella *as the basal-most branch in the angiosperm phylogeny, sampling of more taxa and genes, and further investigations of model specification in phylogenetic analyses are needed before this issue is fully resolved [see discussion in [18,23]].

Several earlier molecular phylogenetic studies based on one or a few genes [27,56,63] did not support the monophyly of magnoliids. Furthermore, morphological studies of angiosperms failed to detect any synapomorphies for this group. The circumscription, monophyly, and relationships of magnoliids has only recently been established based on phylogenetic analyses of multiple genes [14,15]. These earlier multigene trees provided only weak to moderate support for the monophyly of magnoliids and the sister group relationships of the Canellales/Piperales and Laurales/Magnoliales. A recent study using eight plastid, mitochondrial, and nuclear genes [17] provided the first strong support for both the monophyly and relationships among the four orders of magnoliids. Our phylogenetic trees based on 61 plastid protein-coding genes also provide strong support for the monophyly of magnoliids and the sister relationship between the Canellales/Piperales and Laurales/Magnoliales.

One of the most controversial remaining issues regarding relationships among angiosperms concerns the resolution of relationships among the magnoliids, monocots and eudicots. Previous phylogenetic studies have supported three different hypotheses of relationships among these lineages: (1) (magnoliids (monocots, eudicots)), (2) (monocots (magnoliids, eudicots)), and (3) (eudicots (magnoliids, monocots)). The first hypothesis was supported in phylogenetic analyses based on phytochrome genes [3] and 17 plastid genes [6] but bootstrap support for a sister relationship of monocots and eudicots was only 67%. Several studies supported the second hypothesis [7,16,68], however, bootstrap support was again weak ranging from 55 – 78%. The three-gene phylogenetic tree of Soltis et al. [27] supported the third hypothesis with only 56% jackknife support. This relationship was also recovered in a *matK *gene tree with a parsimony bootstrap value of 78% and a posterior probability of 0.73 [29]. Both MP and ML trees based on 61 plastid-encoded protein genes support hypothesis 1 (Figs. 8, 9). Branch support for this hypothesis is moderate (MP, Fig. 8) or strong (ML, Fig. 9). Congruence of the results from both MP and ML analyses is notable because our previous phylogenetic analyses using whole plastid genomes that included only one member of the magnoliid clade (*Calycanthus*, [18,67]) were incongruent. In these earlier studies, MP trees supported hypothesis 2 (monocots sister to a clade that included magnoliids and eudicots), whereas ML trees supported hypothesis 1 (magnoliids sister to a clade that included monocots and dicots). These differences provide yet another example of the importance of expanded taxon sampling in phylogenetic studies using sequences from whole plastid genomes [18,67]. The addition of other angiosperm lineages, especially members of the Chloranthales, Certatophyllaceae, and Illiciales may be critical for providing additional resolution of relationships among the major clades.

## Conclusion

The genome sequences of three additional magnoliids have a very similar size and organization to the ancestral angiosperm plastid genome. Comparisons of 34 seed plant plastid genomes confirm that GC content is unevenly distributed across the genome by location, codon position, and functional group. Phylogenetic analyses for 61 protein-coding genes using both maximum parsimony and maximum likelihood methods for 35 seed plants provide moderate or strong support for the placement of *Amborella *sister to all other angiosperms. Furthermore, there is strong support for the monophyly of magnoliids and for the recognition of two major clades, the Canellales/Piperales and the Laurales/Magnoliales. Finally, phylogenetic analyses provide the strongest support so far that magnoliids are sister to a large clade that includes both monocots and eudicots.

## Methods

### Plastid isolation, amplification, and sequencing

10–20 g of fresh leaf material of *Drimys granatenis *and *Piper coenoclatum *was used for the plastid isolation. Leaf material was obtained from the University of Connecticut Greenhouses (accession numbers 200100052 for *Drimys *and 199600027 for *Piper*). Plastids were isolated from fresh leaves using the sucrose-gradient method [69]. They were then lysed and the entire plastid genome was amplified using Rolling Circular Amplification (RCA, using the REPLI-g^™ ^whole genome amplification kit, Molecular Staging) following the methods outlined in Jansen *et al*. [70]. The RCA product was then digested with the restriction enzymes *Eco*RI and *Bst*BI and the resulting fragments were separated by agarose gel electrophoresis to determine the quality of plastid DNA. The RCA product was sheared by serial passage through a narrow aperture using a Hydroshear device (Gene Machines), and the resulting fragments were enzymatically repaired to blunt ends and gel purified, then ligated into pUC18 plasmids. The clones were introduced into *E. coli *by electroporation, plated onto nutrient agar with antibiotic selection, and grown overnight. Colonies were randomly selected and robotically processed through RCA of plasmid clones, sequencing reactions using BigDye chemistry (Applied Biosystems), reaction cleanup using solid-phase reversible immobilization, and sequencing using an ABI 3730 XL automated DNA sequencer. Detailed protocols are available at [71].

### Genome assembly and annotation

*Drimys and Piper*. Sequences from randomly chosen clones were processed using PHRED and assembled based on overlapping sequence into a draft genome sequence using PHRAP [72]. Quality of the sequence and assembly was verified using Consed [73]. In most regions of the genomes we had 6–12-fold coverage but there were a few areas with gaps or low depth of coverage. PCR and sequencing at the University of Texas at Austin were used to bridge gaps and fill in areas of low coverage in the genome. Additional sequences were added until a completely contiguous consensus was created representing the entire plastid genome with a minimum of 2X coverage and a consensus quality score of Q40 or greater.

*Liriodendron*. The *Liriodendron *plastid genome sequence was obtained using 454 sequencing technology [31]. This work will be described more fully in a separate paper focused in the utility of 454 technology for plastid genome sequencing (J. E. Carlson et al. in progress). Briefly, plastid genome containing clones were identified from a genomic bacterial artificial chromosome (BAC) library that had been constructed for *Liriodendron *[74]. The entire plastid genome was sequenced from a pooled sample of DNAs isolated from three overlapping large-insert BAC clones. The BAC DNAs were processed for 454 sequencing according to protocols provided by 454 Life Sciences (Branford, CT, also see [31]). One-half plate run on the Genome Sequencer 20^™ ^System (Life Sciences, Branford, CT; Roche Applied Science, Indianapolis, IN) generated over 15,000 sequences with an average read length of 103 bp. Reads including vector sequence were removed and the remaining reads were assembled using the 454-de novo assembly software. Each base in the assembly was inferred based on an average of 90 independent reads.

### Genome annotation

The coordinate of each genome was standardized for gene annotation to be the first bp after IRa on the *psbA *side. The genomes were annotated using the program DOGMA (Dual Organellar GenoMe Annotator [75]). All genes, rRNAs, and tRNAs were identified using the plastid/bacterial genetic code.

### Examination of GC content

GC content was calculated for 34 seed plant plastid genomes, including the gymnosperm *Pinus *and 33 angiosperms. GC content was also determined for 66 protein-coding genes. These genes were partitioned into three functional groups (photosynthesis (33), genetic system genes (22), and NADH (11) genes), and GC content was calculated for the entire gene and the first, second, and third codon positions. The genes included in each of these three groups are: (1) photosynthetic genes (*atpA, atpB, atpE, atpF, atpH, atpI, psbZ, petA, petB, petD, petG, petL, petN, psaA, psaB, psaC, psaI, psaJ, psbA, psbB, psbC, psbD, psbE, psbF, psbH, psbI, psbJ, psbK, psbL, psbM, psbN, psbT, rbcL*); (2) genetic system genes (*rpl14, rpl16, rpl2, rpl20, rpl32, rpl33, rpl36, rpoA, rpoB, rpoC1, rpoC2, rps11, rps12, rps14, rps15, rps18, rps19, rps2, rps3, rps4, rps7, rps8*); and (3) NADH genes (*ndhA, ndhB, ndhC, ndhD, ndhE, ndhF, ndhG, ndhH, ndhI, ndhJ, ndhK*). GC content was also plotted over the entire genome for all 34 taxa, which were classified into 10 groups based on gene order and phylogenetic placement (Figs. 8, 9).

Statistical analyses of GC content between rRNA genes and complete genomes and between 72 protein-coding genes and complete genomes were performed using a t test. Analyses of GC content among the three codon positions and among the three functional classes were performed using one way ANOVA. All statistical analyses used the program SPSS, version 9.0 [76].

### Phylogenetic analysis

#### Alignment

The 61 protein-coding genes included in the analyses of Goremykin et al. [9,10] and Leebens-Mack et al. [18] were extracted from *Drimys*, *Liriodendron *and *Piper *using the organellar genome annotation program DOGMA [75]. The same set of 61 genes was extracted from plastid genome sequences of 32 other sequenced genomes (see Table 1 for complete list). All 61 protein-coding genes of the 35 taxa were translated into amino acid sequences, which were aligned using MUSCLE [77] followed by manual adjustments, and then nucleotide sequences of these genes were aligned by constraining them to the aligned amino acid sequences. A Nexus file with character sets for phylogenetic analyses was generated after nucleotide sequence alignment was completed. The complete nucleotide alignment is deposited online at [78].

#### Tree reconstruction

Phylogenetic analyses using maximum parsimony (MP) and maximum likelihood (ML) were performed using PAUP* version 4.0b10 [79] on a data including 35 taxa (Table 1). Phylogenetic analyses excluded gap regions. All MP searches included 100 random addition replicates and TBR branch swapping with the Multrees option. Modeltest 3.7 [80] was used to determine the most appropriate model of DNA sequence evolution for the combined 61-gene dataset. Hierarchical likelihood ratio tests and the Akaikle information criterion were used to assess which of the 56 models best fit the data, which was determined to be GTR + I + Γ by both criteria. For ML analyses we performed an initial parsimony search with 100 random addition sequence replicates and TBR branch swapping, which resulted in a single tree. Model parameters were optimized onto the parsimony tree. We fixed these parameters and performed a ML analysis with three random addition sequence replicates and TBR branch swapping. The resulting ML tree was used to re-optimize model parameters, which then were fixed for another ML search with three random addition sequence replicates and TBR branch swapping. This successive approximation procedure was repeated until the same tree topology and model parameters were recovered in multiple, consecutive iterations. This tree was accepted as the final ML tree (Fig. 9). Successive approximation has been shown to perform as well as full-optimization analyses for a number of empirical and simulated datasets [81]. Non-parametric bootstrap analyses [82] were performed for MP analyses with 1000 replicates with TBR branch swapping, 1 random addition replicate, and the Multrees option and for ML analyses with 100 replicates with NNI branch swapping, 1 random addition replicate, and the Multrees option.

#### Test of alternate topology

A Shimodaira-Hasegawa (SH) test [32] was performed to determine if the alternative topology with *Amborella *+ Nymphaeales basal was significantly worse than the ML tree that places *Amborella *alone as the basal angiosperm lineage. A constraint topology with this alternative tree topology was used and the SH test was conducted using RELL optimization [83] as implemented in PAUP* version 4.0b10 [79].

## Abbreviations

IR inverted repeat; SSC, small single copy; LSC, large single copy, bp, base pair; ycf, hypothetical chloroplast reading frame; rrn, ribosomal RNA; MP, maximum parsimony; ML, maximum likelihood.

## Authors' contributions

CZ finished and annotated the *Drimys *and *Piper *plastid genomes, wrote perl scripts to automate the alignment of both amino acid and nucleotide sequences, performed comparisons of GC content, and wrote several sections of the manuscript; CP assisted in plastid isolations and rolling circular amplification of the *Drimys *and *Piper *genomes, assisted in finishing the genome sequences, and extracting gene sequences; JVK and JLB generated libraries, performed the draft genome sequencing and assembly of *Drimys *and *Piper*; RKJ assisted with plastid isolations, finishing, and annotation of the *Drimys *and *Piper *genomes, performed phylogenetic analyses, and wrote several portions of the manuscript; JLM, JC, and CWP sequenced, annotated, and extracted gene sequences from the *Liriodendron *plastid genome. All authors have read and approved the final manuscript.

**Table 2:** Taxa included in phylogenetic analyses with GenBank accession numbers and references.

Taxon	GenBank Accession Numbers	Reference
Gymnosperms – Outgroups		
*Pinus thunbergii*	NC_001631	Wakasugi et al. 1994 [84]
*Ginkgo biloba*	DQ069337-DQ069702	Leebens-Mack et al 2005 [18]
		
Basal Angiosperms		
*Amborella trichopoda*	NC_005086	Goremykin et al. 2003 [9]
*Nuphar advena*	DQ069337-DQ069702	Leebens-Mack et al 2005 [18]
*Nymphaea alba*	NC_006050	Goremykin et al. 2004 [10]
		
Magnoliids		
*Calycanthus floridus*	NC_004993	Goremykin et al. 2003 [24]
*Drimys granatensis*	DQ887676	Current study
*Liriodendron tulipifera*	NC_008326	Current study
*Piper coenoclatum*	DQ887677	Current study
		
Monocots		
*Acorus americanus*	DQ069337-DQ069702	Leebens-Mack et al 2005 [18]
*Oryza sativa*	NC_001320	Hiratsuka et al. 1989 [85]
*Phalaenopsis aphrodite*	NC_007499	Chang et al. 2006 [19]
*Saccharum officinarum*	NC_006084	Asano et al. 2004 [86]
*Triticum aestivum*	NC_002762	Ikeo and Ogihara, unpublished
*Typha latifolia*	DQ069337-DQ069702	Leebens-Mack et al 2005 [18]
*Yucca schidigera*	DQ069337-DQ069702	Leebens-Mack et al 2005 [18]
*Zea mays*	NC_001666	Maier et al. 1995 [42]
		
Eudicots		
*Arabidopsis thaliana*	NC_000932	Sato et al. 1999 [43]
*Atropa belladonna*	NC_004561	Schmitz-Linneweber et al. 2002 [87]
*Citrus sinensis*	NC_008334	Bausher et al. 2006 [88]
*Cucumis sativus*	NC_007144	Plader et al. unpublished
*Eucalyptus globulus*	NC_008115	Steane 2005 [39]
*Glycine max*	NC_007942	Saski et al. 2005 [89]
*Gossypium hirsutum*	NC_007944	Lee et al. 2006 [90]
*Lotus corniculatus*	NC_002694	Kato et al. 2000 [44]
*Medicago truncatula*	NC_003119	Lin et al., unpublished
*Nicotiana tabacum*	NC_001879	Shinozaki et al. 1986 [91]
*Oenothera elata*	NC_002693	Hupfer et al. 2000 [92]
*Panax schinseng*	NC_006290	Kim and Lee 2004 [45]
*Populus trichocarpa*	NC_008235	unpublished
*Ranunculus macranthus*	DQ069337-DQ069702	Leebens-Mack et al 2005 [18]
*Solanum lycopersicum*	DQ347959	Daniell et al. 2006 [47]
*Solanum bulbocastanum*	NC_007943	Daniell et al. 2006 [47]
*Spinacia oleracea*	NC_002202	Schmitz-Linneweber et al. 2001 [38]
*Vitis vinifera*	NC_007957	Jansen et al. 2006 [67]

## References

[B1] Darwin C, Darwin F, Seward AC (1903). Letter to J. D. Hooker. More letters of Charles Darwin.

[B2] Donoghue DJ, Mathews S (1998). Duplicate genes and the root of angiosperms, with an example using phytochrome sequences. Mol Phylogen Evol.

[B3] Mathews S, Donoghue MJ (1999). The root of angiosperm phylogeny inferred from duplicate phytochrome genes. Science.

[B4] Barkman TJ, Chenery G, McNeal JR, Lyons-Weiler J, Ellisens WJ, Moore G, Wolfe AD, dePamphilis CW (2000). Independent and combined analyses of sequences from all three genomic compartments converge on the root of flowering plant phylogeny. Proc Natl Acad Sci USA.

[B5] Doyle JA, Endress PK (2000). Morphological phylogenetic analysis of basal angiosperms: Comparison and combination with molecular data. Int J Plt Sci.

[B6] Graham SW, Olmstead RG (2000). Utility of 17 chloroplast genes for inferring the phylogeny of the basal angiosperms. Am J Bot.

[B7] Zanis MJ, Soltis DE, Soltis PS, Mathews S, Donoghue MJ (2002). The root of the angiosperms revisited. Proc Natl Acad Sci USA.

[B8] Borsch T, Hilu KW, Quandt DV, Wilde Neinhuis C, Barthlott W (2003). Noncoding plastid trnT-*trnF *sequences reveal a well-resolved phylogeny of basal angiosperms. J Evol Biol.

[B9] Goremykin VV, Hirsch-Ernst KI, Wolfl S, Hellwig FH (2003). Analysis of the *Amborella trichopoda *chloroplast genome sequence suggests that *Amborella *is not a basal angiosperm. Mol Biol Evol.

[B10] Goremykin VV, Hirsch-Ernst KI, Wolfl S, Hellwig FH (2004). The chloroplast genome of *Nymphaea alba*: whole-genome analyses and the problem of identifying the most basal angiosperm. Mol Biol Evol.

[B11] Goremykin VV, Holland B, Hirsch-Ernst KI, Hellwig FH (2005). Analysis of *Acorus calamus *chloroplast genome and its phylogenetic implications. Mol Biol Evol.

[B12] Davies TJ, Barraclough TG, Chase MW, Soltis PS, Soltis DE, Savolainen V (2004). Darwin's abominable mystery: insights from a supertree of the angiosperms. Proc Natl Acad Sci USA.

[B13] Soltis DE, Soltis PS (2004). *Amborella *not a "basal angiosperm"? Not so fast. Amer J Bot.

[B14] Qiu Y-L, Lee J, Bernasconi-Quadroni F, Soltis DE, Soltis PS, Zanis M, Zimmer EA, Chen Z, Savolainen V, Chase MW (1999). The earliest angiosperms: evidence from mitochondrial, plastid and nuclear genomes. Nature.

[B15] Qiu Y-L, Lee J, Bernasconi-Quadroni F, Soltis DE, Soltis PS, Zanis M, Zimmer EA, Chen Z, Savolainen V, Chase MW (2000). Phylogeny of basal angiosperms: analyses of five genes from three genomes. Int J Plt Sci.

[B16] Qiu Y-L, Dombrovska O, Lee J, Li L, Whitlock BA, Bernasconi-Quadroni F, Rest JS, Davis CC, Borsch T, Hilu KW, Renner SS, Soltis DE, Soltis PS, Zanis MJ, Cannone JJ, Gutell RR, Powell M, Savolainen V, Chatrou LW, Chase MW (2005). Phylogenetic analysis of basal angiosperms based on nine plastid, mitochondrial, and nuclear genes. Int J Plt Sci.

[B17] Qiu Y-L, Li L, Hendry T, Li R, Taylor DW, Issa MJ, Ronen AJ, Vekaria ML, White AM (2006). Reconstructing the basal angiosperm phylogeny: evaluating information content of the mitochondrial genes. Taxon.

[B18] Leebens-Mack J, Raubeson LA, Cui L, Kuehl J, Fourcade M, Chumley T, Boore JL, Jansen RK, dePamphilis CW (2005). Identifying the basal angiosperms in chloroplast genome phylogenies: sampling one's way out of the Felsenstein zone. Mol Biol Evol.

[B19] Chang C-C, Lin H-C, Lin I-P, Chow T-Y, Chen H-H, Chen W-H, Cheng C-H, Lin C-Y, Liu S-M, Chang C-C, Chaw S-M (2006). The chloroplast genome of *Phalaenopsis aphrodite *(Orchidaceae): comparative analysis of evolutionary rate with that of grasses and its phylogenetic implications. Mol Biol Evol.

[B20] Stefanovic S, Rice DW, Palmer JD (2004). Long branch attraction, taxon sampling, and the earliest angiosperms: *Amborella *or monocots?. BMC Evol Biol.

[B21] Martin W, Deusch O, Stawski N, Grunheit N, Goremykin V (2005). Chloroplast genome phylogenetics: why we need independent approaches to plant molecular evolution. Trends Plant Sci.

[B22] Soltis DE, Albert VA, Savolainen V, Hilu K, Qiu Y-Q, Chase MW, Farris JS, Stefanović S, Rice DW, Palmer JD, Soltis PS (2004). Genome-scale data, angiosperm relationships, and 'ending incongruence': a cautionary tale in phylogenetics. Trends Plant Sci.

[B23] Lockhart PJ, Penny D (2005). The place of *Amborella *within the radiation of angiosperms. Trends Plant Sci.

[B24] Goremykin VV, Hirsch-Ernst KI, Wolfl S, Hellwig FH (2003). The chloroplast genome of the "basal" angiosperm *Calycanthus fertilis *– structural and phylogenetic analyses. Plt Syst Evol.

[B25] Soltis DE, Soltis PS, Endress PK, Chase MW (2005). Phylogeny and evolution of Angiosperms.

[B26] Soltis PS, Soltis DE, Chase MW (1999). Angiosperm phylogeny inferred from multiple genes as a tool for comparative biology. Nature.

[B27] Soltis DE, Soltis PS, Chase MW, Mort ME, Albach DC, Zanis M, Savolainen V, Hahn WJ, Hoot SB, Fay MF, Axtell M, Swensen SM, Prince LM, Kress WJ, Nixon KC, Farris JS (2000). Angiosperm phylogeny inferred from 18S rDNA, *rbcL*, and *atpB *sequences. Bot J Linn Soc.

[B28] Zanis MJ, Soltis PS, Qiu Y-L, Zimmer EA, Soltis DE (2003). Phylogenetic analyses and perianth evolution in basal angiosperms. Ann Missouri Bot Gard.

[B29] Hilu KW, Borsch T, Muller K, Soltis DE, Soltis PS, Savolainen V, Chase M, Powell M, Alice L, Evans R, Sauquet H, Neinhuis C, Slotta T, Rohwer J, Chatrou L (2003). Inference of angiosperm phylogeny based on *matK *sequence information. Amer J Bot.

[B30] Kim S, Koh J, Yoo MJ, Kong H, Hu Y, Ma H, Soltis PS, Soltis DE (2005). Expression of floral MADS-box genes in basal angiosperms: implications for the evolution of floral regulators. Plant J.

[B31] Margulies M, Egholm M, Altman WE, Attiya S, Bader JS, Bemben LA, Berka J, Braverman MS, Chen YJ, Chen Z, Dewell SB, Du L, Fierro JM, Gomes XV, Godwin BC, He W, Helgesen S, Ho CH, Irzyk GP, Jando SC, Alenquer ML, Jarvie TP, Jirage KB, Kim JB, Knight JR, Lanza JR, Leamon JH, Lefkowitz SM, Lei M, Li J, Lohman KL, Lu H, Makhijani VB, McDade KE, McKenna MP, Myers EW, Nickerson E, Nobile JR, Plant R, Puc BP, Ronan MT, Roth GT, Sarkis GJ, Simons JF, Simpson JW, Srinivasan M, Tartaro KR, Tomasz A, Vogt KA, Volkmer GA, Wang SH, Wang Y, Weiner MP, Yu P, Begley RF, Rothberg JM (2005). Genome sequencing in microfabricated high-density picolitre reactors. Nature.

[B32] Shimodaira H, Hasegawa M (1999). Multiple comparisons of log-likelihoods with applications to phylogenetic inference. Mol Biol Evol.

[B33] Raubeson LA, Jansen RK, Henry H (2005). Chloroplast genomes of plants. Diversity and Evolution of Plants-Genotypic and Phenotypic Variation in Higher Plants.

[B34] Goulding SE, Olmstead RG, Morden CW, Wolfe KH (1996). Ebb and flow of the chloroplast inverted repeat. Mol Gen Gen.

[B35] Sugiura C, Kobayashi Y, Aoki S, Sugita C, Sugita M (2003). Complete chloroplast DNA sequence of the moss *Physcomitrella patens*: evidence for the loss and relocation of *rpoA *from the chloroplast to the nucleus. Nucl Acids Res.

[B36] Palmer JD, Nugent JM, Herbon LA (1987). Unusual structure of geranium chloroplast DNA – A triple-sized inverted repeat, extensive gene duplications, multiple inversions, and 2 repeat families. Proc Natl Acad Sci USA.

[B37] Chumley TW, Palmer JD, Mower JP, Fourcade HM, Caile PJ, Boore JL, Jansen RK The complete chloroplast genome sequence of *Pelargonium × hortorum*: Organization and evolution of the largest and most highly rearranged chloroplast genome of land plants. Mol Biol Evol.

[B38] Schmitz-Linneweber C, Maier RM, Alcaraz JP, Cottet A, Herrmann RG, Mache R (2001). The plastid chromosome of spinach (*Spinacia oleracea*): complete nucleotide sequence and gene organization. Plt Mol Biol.

[B39] Steane DA (2005). Complete nucleotide sequence of the chloroplast genome from the Tasmanian blue gum, *Eucalyptus globulus *(Myrtaceae). DNA Res.

[B40] Ohtani K, Yamamoto H, Akimitsu K (2002). Sensitivity to *Alternaria alternata *toxin in citrus because of altered mitochondrial RNA processing. Proc Natl Acad Sci USA.

[B41] Shimada H, Sugiura M (1991). Fine structural features of the chloroplast genome: comparison of the sequenced chloroplast genomes. Nucl Acids Res.

[B42] Maier RM, Neckermann K, Igloi GL, Kossel H (1995). Complete sequence of the maize chloroplast genome: gene content, hotspots of divergence and fine tuning of genetic information by transcript editing. J Mol Biol.

[B43] Sato S, Nakamura Y, Kaneko T, Asamizu E, Tabata S (1999). Complete structure of the chloroplast genome of *Arabidopsis thaliana*. DNA Res.

[B44] Kato T, Kaneko T, Sato S, Nakamura Y, Tabata S (2000). Complete structure of the chloroplast genome of a legume, *Lotus japonicus*. DNA Res.

[B45] Kim K-J, Lee H-L (2004). Complete chloroplast genome sequence from Korean Ginseng (*Panax schiseng *Nees) and comparative analysis of sequence evolution among 17 vascular plants. DNA Res.

[B46] Chaw S-M, Chang C-C, Chen H-L, Li W-H (2004). Dating the monocot-dicot divergence and the origin of core eudicots using whole chloroplast genomes. J Mol Evol.

[B47] Daniell H, Lee S-B, Grevich J, Saksi C, Quesada-Vargas T, Guda C, Tomkins J, Jansen RK (2006). Complete chloroplast genome sequences of *Solanum bulbocastanum*, *Solanum lycopersicum *and comparative analyses with other Solanaceae genomes. Theor Appl Genet.

[B48] Liu Q, Xue Q (2005). Comparative studies on codon usage pattern of chloroplasts and their host nuclear genes in four plant species. J Genet.

[B49] Morton BR (1993). Chloroplast DNA codon use: Evidence for selection at the *psbA *locus based on tRNA availability. J Mol Evol.

[B50] Morton BR (1994). Codon use and the rate of divergence of land plant chloroplast genes. Mol Biol Evol.

[B51] Morton BR (1998). Selection on the codon bias of chloroplast and cyanelle genes in different plant and algal lineages. J Mol Evol.

[B52] Morton BR, Levin JA (1997). The atypical codon usage of the plant *psbA *gene maybe the remnant of an ancestral bias. Proc Natl Acad Sci USA.

[B53] Wall DP, Herbeck JT (2003). Evolutionary patterns of codon usage in the chloroplast gene *rbcL*. J Mol Evol.

[B54] Martin PG, Dowd JM (1991). Studies of angiosperm phylogeny using protein sequences. Ann Misssouri Bot Gard.

[B55] Hamby RK, Zimmer EA, Soltis PS, Soltis DE, Doyle JJ (1992). Ribosomal RNA as a phylogenetic tool in plant systematics. Molecular Systematics of Plants.

[B56] Chase M, Soltis D, Olmstead R, Morgan D, Les D, Mishler B, Duvall M, Price R, Hills H, Qui Y-L, Kron K, Rettig J, Conti E, Palmer J, Manhart J, Sytsma K, Michaels H, Kress J, Karol K, Clark D, Hedren M, Gaut B, Jansen R, Kim K-J, Wimpee C, Smith J, Furnier G, Straus S, Xiang Q-Y, Plunkett G, Soltis P, Swensen S, Williams S, Gadek P, Quinn C, Equiarte L, Golenberg E, Learn G, Graham S, Barrett S, Dayanandan S, Albert V (1993). Phylogenetics of seed plants: an analysis of nucleotide sequences from the plastid gene *rbc*L. Ann Missouri Bot Gard.

[B57] Qiu Y-L, Chase MW, Les DH, Parks CR (1993). Molecular phylogenetics of the Magnoliidae: cladistic analyses of nucleotide sequences of the plastid gene *rbcL*. Ann Missouri Bot Gard.

[B58] Qiu Y-L, Lee J, Whitlock BA, Bernasconi-Quadroni F, Dombrovska O (2001). Was the ANITA rooting of the angiosperm phylogeny affected by long branch attraction?. Mol Biol Evol.

[B59] Soltis DE, Soltis PS, Nickrent DL, Johnson LA, Hahn WJ, Hoot SB, Sweere JA, Kuzoff RK, Kron KA, Chase M, Swensen SM, Zimmer E, Shaw SM, Gillespie LJ, Kress WJ, Sytsma K (1997). Angiosperm phylogeny inferred from 18S ribosomal DNA sequences. Ann Missouri Bot Gard.

[B60] Hoot SB, Magallon S, Crane PR (1999). Phylogeny of basal tricolpates based on three molecular data sets: *atpB*, *rbcL*, and 18S nuclear ribosomal DNA sequences. Ann Missouri Bot Gard.

[B61] Mathews S, Donoghue MJ (2000). Basal angiosperm phylogeny inferred from duplicated phytochromes A and C. Int J Plt Sci.

[B62] Parkinson CL, Adams KL, Palmer JD (1999). Multigene analyses identify the three earliest lineages of extant flowering plants. Curr Biol.

[B63] Savolainen V, Chase MW, Morton CM, Soltis DE, Bayer C, Fay MF, De Bruijn A, Sullivan S, Qiu Y-L (2000). Phylogenetics of flowering plants based upon a combined analysis of plastid *atpB *and *rbcL *gene sequences. Syst Biol.

[B64] Aoki S, Uehara K, Imafuku M, Hasebe M, Ito M (2004). Phylogeny and divergence of basal angiosperms inferred from *APETALA3*- and *PISTILLATA*-like MADS-box genes. J Plt Res.

[B65] Kim ST, Yoo MJ, Albert VA, Farris JS, Soltis PS, Soltis DE (2004). Phylogeny and diversification of B-function MADS-box genes in angiosperms: Evolutionary and functional implications of a 260-million-year-old duplication. Amer J Bot.

[B66] Löhne C, Borsch T (2005). Molecular evolution and phylogenetic utility of the *petD *group II intron: a case study in basal angiosperms. Mol Biol Evol.

[B67] Jansen RK, Kaittanis C, Saski C, Lee SB, Tomkins J, Alverson AJ, Daniell H (2006). Phylogenetic analyses of *Vitis *(*Vitaceae*) based on complete chloroplast genome sequences: effects of taxon sampling and phylogenetic methods on resolving relationships among rosids. BMC Evol Biol.

[B68] Nickrent DL, Blarer A, Qiu Y-L, Soltis DE, Soltis PS, Zanis M (2002). Molecular data place Hydnoraceae with Aristolochiaceae. Amer J Bot.

[B69] Palmer JD, Weissbach A, Weissbach H (1986). Isolation and structural analysis of chloroplast DNA. Methods Enzymol.

[B70] Jansen RK, Raubeson LA, Boore JL, dePamphilis CW, Chumley TW, Haberle RC, Wyman SK, Alverson AJ, Peery R, Herman SJ, Fourcade HM, Kuehl JV, McNeal JR, Leebens-Mack J, Cui L (2005). Methods for obtaining and analyzing chloroplast genome sequences. Meth Enzym.

[B71] DOE Joint Genome Institute sequencing protocols. http://www.jgi.doe.gov/sequencing/index.html.

[B72] Ewing B, Green P (1998). Base-calling of automated sequencer traces using phred. II. Error probabilities. Genome Res.

[B73] Gordon D, Abajian C, Green P (1998). Consed: a graphical tool for sequence finishing. Genome Res.

[B74] Liang H, Fang EG, Tomkins JP, Luo M, Kudrna D, Arumuganathan K, Zhao S, Schlarbaum SE, Banks JA, Leebens-Mack JH, dePamphilis CW, Mandoli DF, Wing RA, Carlson JE (2006). Development of a BAC library for yellow-poplar (*Liriodendron tulipifera*) and the identification of genes associated with flower development and lignin biosynthesis. Tree Genet Genomes.

[B75] Wyman SK, Boore JL, Jansen RK (2004). Automatic annotation of organellar genomes with DOGMA. Bioinformatics.

[B76] (2001). SPSS for Windows, Rel. 9.0.

[B77] Edgar RC (2004). MUSCLE: multiple sequence alignment with high accuracy and high throughput. Nucl Acids Res.

[B78] Cui L, Veeraraghavan N, Richer A, Wall K, Jansen RK, Leebens-Mack J, Makalowska I, dePamphillis CW (2006). ChloroplastDB: the chloroplast genome database. Nucl Acids Res.

[B79] Swofford DL (2003). PAUP*: Phylogenetic analysis using parsimony (* and other methods), ver 40.

[B80] Posada D, Crandall KA (1998). MODELTEST: testing the model of DNA substitution. Bioinformatics.

[B81] Sullivan J, Abdo Z, Joyce P, Swofford DL (2005). Evaluating the performance of a successive-approximations approach to parameter optimization in maximum-likelihood phylogeny estimation. Mol Biol Evol.

[B82] Felsenstein J (1985). Confidence limits on phylogenies: an approach using the bootstrap. Evolution.

[B83] Goldman N, Anderson JP, Rodrigo AG (2000). Likelihood-based tests of topologies in phylogenetics. Syst Biol.

[B84] Wakasugi T, Tsudzuki J, Ito S, Nakashima K, Tsudzuki T, Sugiura M (1994). Loss of all *ndh *genes as determined by sequencing the entire chloroplast genome of the black pine *Pinus thunbergii*. Proc Natl Acad Sci USA.

[B85] Hiratsuka J, Shimada H, Whittier R, Ishibashi T, Sakamoto M, Mori M, Kondo C, Honji Y, Sun CR, Meng BY, Li YQ, Kanno A, Nishizawa Y, Hirai A, Shinozaki K, Sugiura M (1989). The complete sequence of the rice (*Oryza sativa*) chloroplast genome: intermolecular recombination between distinct tRNA genes accounts for a major plastid DNA inversion during the evolution of the cereals. Mol Gen Genet.

[B86] Asano T, Tsudzuki T, Takahashi S, Shimada H, Kadowaki K (2004). Complete nucleotide sequence of the sugarcane (*Saccharum officinarum*) chloroplast genome: a comparative analysis of four monocot chloroplast genomes. DNA Res.

[B87] Schmitz-Linneweber C, Regel R, Du TG, Hupfer H, Herrmann RG, Maier RM (2002). The plastid chromosome of *Atropa belladonna *and its comparison with that of *Nicotiana tabacum*: the role of RNA editing in generating divergence in the process of plant speciation. Mol Biol Evol.

[B88] Bausher MG, Singh ND, Lee S, Jansen RK, Daniell H The complete chloroplast genome sequence of Citrus sinensis (L.) Osbeck var 'Ridge Pineapple': organization and phylogenetic relationships to other angiosperms. BMC Plt Biol.

[B89] Saski C, Lee S, Daniell H, Wood T, Tomkins J, Kim H-G, Jansen RK (2005). Complete chloroplast genome sequence of *Glycine max *and comparative analyses with other legume genomes. Plt Mol Biol.

[B90] Lee S-B, Kaittanis C, Jansen RK, Hostetler JB, Tallon LJ, Town CD, Daniell H (2006). The complete chloroplast genome sequence of *Gossypium hirsutum*: organization and phylogenetic relationships to other angiosperms. BMC Genomics.

[B91] Shinozaki K, Ohme M, Tanaka M, Wakasugi T, Hayashida N, Matsubayashi T, Zaita N, Chunwongse J, Obokata J, Yamaguchi-Shinozaki K, Ohto C, Torazawa K, Meng BY, Sugita M, Deno H, Kamogashira T, Yamada K, Kusuda J, Takaiwa F, Kato A, Tohdoh N, Shimada H, Sugiura M (1986). The complete nucleotide sequence of the tobacco chloroplast genome: its gene organization and expression. EMBO J.

[B92] Hupfer H, Swaitek M, Hornung S, Herrmann RG, Maier RM, Chiu WL, Sears B (2000). Complete nucleotide sequence of the *Oenothera elata *plastid chromosome, representing plastome 1 of the five distinguishable *Euoenthera *plastomes. Mol Gen Genet.

[B93] APG II 2002 (2003). An update of the Angiosperm Phylogeny Group classification for the orders and families of flowering plants: APG II. Bot J Linn Soc.

